# Prediction of health care expenditure increase: how does pharmacotherapy contribute?

**DOI:** 10.1186/s12913-019-4616-x

**Published:** 2019-12-11

**Authors:** Annika M. Jödicke, Urs Zellweger, Ivan T. Tomka, Thomas Neuer, Ivanka Curkovic, Malgorzata Roos, Gerd A. Kullak-Ublick, Hayk Sargsyan, Marco Egbring

**Affiliations:** 1 Department of Clinical Pharmacology and Toxicology, University Hospital Zurich, University of Zurich, Zurich, Switzerland; 2 Swiss Federal Institute of Technology Zurich (ETH Zurich), Zurich, Switzerland; 3 Department of Client Services & Claims, Helsana Group, Zurich, Switzerland; 4 EPha.ch AG, Data Science in Healthcare, Zurich, Switzerland; 5 EBPI, Department of Biostatistics, University of Zurich, Zurich, Switzerland

**Keywords:** Machine learning, Health care utilisation, Health care costs, Boosted decision tree, Neural network, Pharmacology, Claims data

## Abstract

**Background:**

Rising health care costs are a major public health issue. Thus, accurately predicting future costs and understanding which factors contribute to increases in health care expenditures are important. The objective of this project was to predict patients healthcare costs development in the subsequent year and to identify factors contributing to this prediction, with a particular focus on the role of pharmacotherapy.

**Methods:**

We used 2014–2015 Swiss health insurance claims data on 373′264 adult patients to classify individuals’ changes in health care costs. We performed extensive feature generation and developed predictive models using logistic regression, boosted decision trees and neural networks. Based on the decision tree model, we performed a detailed feature importance analysis and subgroup analysis, with an emphasis on drug classes.

**Results:**

The boosted decision tree model achieved an overall accuracy of 67.6% and an area under the curve-score of 0.74; the neural network and logistic regression models performed 0.4 and 1.9% worse, respectively. Feature engineering played a key role in capturing temporal patterns in the data. The number of features was reduced from 747 to 36 with only a 0.5% loss in the accuracy. In addition to hospitalisation and outpatient physician visits, 6 drug classes and the mode of drug administration were among the most important features. Patient subgroups with a high probability of increase (up to 88%) and decrease (up to 92%) were identified.

**Conclusions:**

Pharmacotherapy provides important information for predicting cost increases in the total population. Moreover, its relative importance increases in combination with other features, including health care utilisation.

## Introduction

Rising health care costs are a major economic and public health issue worldwide [1, 2]: According to the World Health Organization, health care accounted for 7.9% of Europe’s gross domestic product (GDP) in 2015 [3]. In Switzerland, the health care sector contributes substantially to the national GDP, and has increased from 10.7 to 12.1% between 2010 and 2015 [3]. Moreover, because health care utilisation costs may serve as a surrogate for an individual’s health status [4], understanding which factors contribute to increases in health expenditures may provide insight into risk factors and potential starting points for preventive measures.

Several studies [4–21] have addressed the prediction of health care costs, approaching the issue as either a regression problem or a classification problem (classifying costs into predefined “buckets”). Morid et al. [22] conducted a literature review summarising and comparing the existing models. As far as the annual difference in costs is concerned, we are aware of only 1 study [23], which classified healthcare costs development into only two classes (binary classification). Previous studies also examined a broad variety of features. The most commonly used features include different sets of demographic features, health care utilisation parameters (e.g. hospitalisation or outpatient visits), drug codes, diagnosis codes, procedure codes, various chronic disease scores and cost features.

In this study, we aimed to predict changes in patients’ health care costs in the subsequent year and to identify factors contributing substantially to this prediction. In particular, we focused on the role of pharmacotherapy and other medical features such as hospitalisations and outpatient physician visits. We approached the problem as a binary classification task, predicting whether patient’s total costs would increase or decrease in 2015, based on their characteristics in 2014. We compared the performance of 3 different models: feedforward neural networks (FNN), boosted decision trees (BDT) and logistic regression (LR). To capture different patterns in the data, we performed extensive feature engineering and introduced new domain-specific features, such as the drug administration mode. Finally, we performed a detailed feature importance analysis and subgroup analysis, based on the decision tree model.

## Methods

### Study data

We used anonymised claims data provided by the Helsana Group, one of the largest health insurance companies in Switzerland, which covers about 15% of the population across all regions of the country [24]. Basic health insurance coverage is mandatory in Switzerland. All residents are free to choose their preferred insurance providers, which are privately owned. Insurance coverage is financed by a premium and includes co-payments and deductibles [25]. The amount of the deductible can be chosen by the patient and changed every year. All health care invoices submitted for reimbursement are recorded in Helsana’s claims database [24]. The full dataset comprised information on adults (aged ≥18 years) without additional private insurance. All patients were insured by Helsana throughout the study period (2014–2015), allowing for complete records for both years. Furthermore, we required that all patients had at least 5 drug prescriptions in both calendar years and complete records on all demographic variables. In total, 373′264 patients met these requirements. Our dataset comprised demographic parameters, information on health insurance status, prescribed drugs, claimed health care utilisation, and total costs for each patient. Total costs were defined as gross costs for all invoices submitted for reimbursement, thus not taking co-payments and deductibles into account. Prescribed drugs are displayed using the Global Trade Item Number (GTIN). Additionally, the active component (5th-level Anatomical Therapeutic Chemical (ATC) code [26]) is available for every drug. Diagnoses are not available in our dataset because of legal regulations in Switzerland.

### Introduction of features

Feature engineering plays an important role in most of the machine learning models and can greatly improve prediction accuracy for any task.

Our exploratory linear regression analysis revealed that, compared with the prediction of total costs, the variance of the difference in costs is harder to explain using basic features such as demographics [5, 6, 13–16, 19] or simple count measures [17] described in the literature (Additional file 1: Table S1). Therefore, we performed extensive feature generation to include additional predictors in our models. We assigned names to the feature sets, which we later use to discuss their relative importance for the overall accuracy.

#### Basic features

The included demographic features were age, gender, deductible amount, insurance model and area of residence. We also included the simple count measures of numbers of hospitalisations, outpatient physician office visits, different drugs, and the number of individual prescriptions (GTINs). Because our dataset lacks diagnosis codes, we approximated chronic conditions following the ATC classification proposed by Huber et al. [25] and computed the number of prescribed ATC codes corresponding to each group.

#### Features representing pharmacotherapy

In addition to the derived chronic conditions, we included explicit drug information. To reduce sparsity, we chose 4th-level ATC [26] codes (eg. C01AA, statins) over the 8′705 unique GTINs or the 1′027 5th-level ATC codes. For each of the resulting 449 categories, we computed the number of corresponding prescriptions.

#### Additional features

We included the following additional features: Hospitalisation was identified using Swiss diagnosis-related group (DRG) codes [27]. We generated features displaying the major diagnostic categories derived from DRG codes (e.g., hospitalisation for diseases of the respiratory system), the type of hospital, and the type of harm (e.g., accident, disease), as well as the overall length of hospital stay. To capture temporal patterns [28], we computed the frequencies of outpatient office and bedside visits per month and per quarter of the year. We also included physician’s specialisation, the institution dispensing the drug and the number of visits on weekends (which might indicate acuteness) as features. Additionally, we computed the frequencies of prescriptions for different fine-grained periods of time and the number of prescribed products with certain modes of administration (e.g., intravenous) for each patient. The number of different drug classes and prescriptions (defined as different purchase dates), as well as features representing psychiatric treatment, rehabilitation, nursing home stays, and home care were also included. Finally, we generated a number of descriptive statistics (median, mean, standard deviation, minimum, and maximum) for intervals between, for example, prescriptions, visits and home care to capture a regularity pattern. Our expectation was that, the more regular these events are, the more continuous is the treatment, and that irregularity might point to a more acute condition.

#### Costs feature

Total healthcare costs in 2014 was included only to assess the overall accuracy and to determine whether the medical features provided complementary information.

### Data split

Using random assignment, we divided the dataset into 3 parts: training set (80%), validation set (10%), and test set (10%). The training set was used to develop the prediction models, and the validation set was used for assessing the performance of various methods and for subsequent tuning of the hyperparameters. The test set was reserved for reporting the performance of the final models. We report the basic descriptive statistics in Table 1.

**Table 1:** Study population characteristics (2014)

	All	Train	Validation	Test
Patients, n (%)	373′264 (100%)	298′611 (80%)	37′326 (10%)	37′327 (10%)
Demographics				
Age, median [IQR]	63.8 [49.2, 75.1]	63.8 [49.3, 75.1]	63.6 [49.0, 75.2]	63.8 [49.1, 75.1]
Gender [female], n (%)	226′085 (60.6%)	180′730 (60.5%)	22′565 (60.5%)	22′790 (61.1%)
Language Area, n (%)				
*German*	275′025 (73.7%)	219′998 (73.7%)	27′469 (73.6%)	27′558 (73.8%)
*French*	69′120 (18.5%)	55′292 (18.5%)	6′927 (18.6%)	6′901 (18.5%)
*Italian*	29′119 (7.8%)	23′321 (7.8%)	2′930 (7.8%)	2′868 (7.7%)
Deductible, n (%)				
*CHF 300*	250′287 (67.1%)	200′211 (67.0%)	25′026 (67.0%)	25′050 (67.1%)
*CHF 500–1000*	92′274 (24.7%)	73′900 (24.7%)	9′175 (24.6%)	9′199 (24.6%)
*CHF > 1000*	30′703 (8.2%)	24′500 (8.2%)	3′125 (8.4%)	3′078 (8.2%)
Cost				
Total Costs (CHF), median [IQR]	3′932 [1′944, 8′597]	3′935 [1′946, 8′586]	3′948 [1′958, 8′642]	3′894 [1′915, 8′642]
Cost Difference (CHF)^*^, median [IQR]	93 [−1′746, 2365]	93 [− 1′750, 2′365]	62 [− 1′791, 2′305]	122 [− 1′668, 2′441]
Increase^†^, n (%)	193′766 (51.9%)	155′058 (51.9%)	19′130 (51.3%)	19′578 (52.4%)
Drug Therapy				
Number of drugs^‡^, median [IQR]	9 [6, 15]	9 [6, 15]	9 [6, 15]	9 [6, 15]
Number of prescriptions^§^, median [IQR]	19 [11, 34]	19 [11, 34]	19 [11, 34]	19 [11, 34]
Route of administration, n (%)				
*oral*	369'101 (98.9%)	295′313 (98.9%)	36′926 (98.9%)	36′862 (98.8%)
*intravenous*	122′361 (32.8%)	97′764 (32.7%)	12′427 (33.3%)	12′170 (32.6%)
Health Care Utilisation				
Number of visits^||^, median [IQR]	8 [4, 13]	8 [4, 13]	8 [4, 13]	8 [4, 13]
Hospitalisation [yes], n (%)	66′427 (17.8%)	53′085 (17.8%)	6′688 (17.9%)	6′654 (17.8%)

Descriptive statistics such as median, interquartile range (IQR), absolute and relative frequencies were computed using R (Version 3.3.1). Age was used as 18 age categories in the models, but shown as continuous variable in this table for easier interpretation. ^*^Cost Difference = Total Costs 2015 - Total Costs 2014 (CHF = Swiss Francs), ^†^Increase = Cost Difference > 0, ^‡^Number of different drugs defined by active components, ^§^Number of prescribed drugs, identified by GTIN, ^||^Number of outpatient physician office visits

### Models

We used 3 different methods to develop models for our analysis. As a reference model, we used LR and contrasted its performance to FNN and BDT. All models were developed starting with a set of demographic features. Additional feature sets were added in a stepwise manner, resulting in a total of 747 different features in the complete model (Table 2).

**Table 2:** Comparison of prediction performance of logistic regression (LR), boosted decision tree (BDT) and feedforward neural network (FNN) using different sets of features

		Models					
***Model performance on validation dataset***		LR		BDT		FNN	
**Features**	**Size**	**Acc (%)**	**AUC**	**Acc (%)**	**AUC**	**Acc (%)**	**AUC**
Demographic model*	7	51.2	0.52	51.3	0.52	52.2	0.53
+ number of different drugs	8	58.0	0.61	58.1	0.61	58.7	0.61
+ number of individual prescriptions	8	55.3	0.58	56.9	0.60	57.5	0.60
+ number of hospitalisations	8	61.0	0.62	61.0	0.62	61.1	0.63
+ number of outpatient physician office visits	8	59.4	0.63	60.1	0.63	60.4	0.64
+ chronic conditions	29	54.8	0.57	57.0	0.59	57.5	0.60
Extended model^†^	33	62.8	0.67	63.1	0.68	64.0	0.69
+ additional features	297	64.8	0.70	66.3	0.72	66.1	0.72
+ features representing pharmacotherapy	482	64.5	0.69	65.4	0.71	65.6	0.71
+ total costs	34	62.1	0.67	64.8	0.71	65.7	0.71
+ additional features + total costs	298	65.0	0.70	67.0	0.74	67.0	0.73
Complete model^‡^ without total costs	746	65.3	0.71	66.5	0.73	66.5	0.72
Complete model	747	65.2	0.70	67.4	0.74	67.4	0.73
Backward Deletion	36	–	–	66.9	0.73	–	–
***Model performance on test dataset***							
Complete model^‡^ without total costs	746	65.9	0.71	66.8	0.73	66.4	0.72
Complete model	747	65.7	0.71	67.6	0.74	67.2	0.73
Backward Deletion	36	–	–	67.1	0.73	–	–

Acc = Accuracy, AUC = Area under the curve, Size = Number of features in the model

^*^Demographic model = age + gender + area of residence + deductible + insurance model,

^†^Extended model = Demographic model + number of different drugs + number of individual prescriptions + number of hospitalisations + number of outpatient physician office visits + chronic conditions

^‡^Complete model = Extended model + additional predictors + features representing pharmacotherapy + total costs

Bold data are significant

Because we use BDT (in particular the XgBoost [29] library) extensively for the subsequent analyses, a short overview is in order: BDT is a variant of decision tree methods with a gradient boosting algorithm governing the learning process. In decision trees, the input is mapped to a target label by a recursive creation of decision rules [30], which can be represented as nodes in a graphical tree model. The gradient boosting method produces a prediction model in the form of a weighted average of several weak predictors (decision trees).

### Feature importance analysis using BDT

We used BDT to conduct detailed feature and drug-importance analyses. Using BDT, decision rules can be mapped into respective cuts in our feature space, generating subgroups of patients with a high probability of an increase in costs. In particular, we were interested in medically relevant subgroups, with a particular emphasis on pharmacotherapy.

#### General feature importance

We used backward deletion to assess the general feature importance. Backward deletion begins with all candidate features (here, the complete model), and the deletion of each feature is tested using a chosen model fit criterion. The feature that makes the most statistically insignificant contribution to the model fit quality is deleted. The process is repeated until no further variables can be deleted without a large loss in accuracy. This process is displayed in Additional file 1: Figure S1 in the supplement.

#### Drug importance analysis

##### Conditional drug probabilities

The feature importance analysis based on backward deletion selects features according to their overall contribution to the total accuracy. As the latter depends on the feature’s frequency in the dataset and its relative discriminative contribution, more frequently prescribed drugs have an advantage over those that are prescribed less frequently, even if discriminating less efficiently. In order to get additional insight into the drug-importance, we computed the probability of increase, conditioned on the drug classes and stratified by hospitalisation.

##### Weight analysis

Although conditional drug probabilities provide an important overview, interactions of the drug classes with other features (except for hospitalisation) could not be assessed. Therefore, we performed a weight analysis to investigate the decision tree model predictions using the test set. To understand the concept of weight analysis, it is important to clarify how the BDT prediction is generated during the inference stage. For a given input sample, the BDT maps every feature in the sample to learned weights or scores. The individual score can be either positive or negative, depending on whether the feature contributes to increase or decrease prediction, respectively. The final prediction is an increase, if the sum of all scores is positive; otherwise it is a decrease. Thus, by analysing the weights of particular features using a sample of inputs, one can understand how often and how strongly these features contribute [31]. Using this intuition, we filtered out the drug classes that contributed to increases or decreases with a high proportion (at least 5% of the overall positive or negative score).

##### Subgroup analysis

BDT produces a prediction model in the form of a weighted average of several weak predictors. To find examples of highly predictive subgroups involving drug classes, we employed the following strategy: First, we filtered out all decision paths in all trees where a particular drug class was used. More precisely, we considered only the paths where the prescription of the drug contributed. Next, we measured the conditional probability of increase for the cuts given by the filtered paths. We denote this probability by *P(increase | cut).* For every such a cut, we computed the conditional probability without the drug class cut*, P(increase | cut without drug class).* We defined a *gain* to be the *difference |P(increase | cut) - P(increase | cut without drug class)|.* Lastly, we chose the subgroups with high values of *gain.*

## Results

In Table 1 we show the basic descriptive statistics for the total dataset, as well as for the three subsets. As one can see from the table, the training, validation and test datasets follow the same distribution over all parameters. In particular, it is important that the variation of the annual cost difference and the proportion of cost increase/decrease is small (within ±0.6% for the cost increase).

### Performance of models

**Fig. 1:**
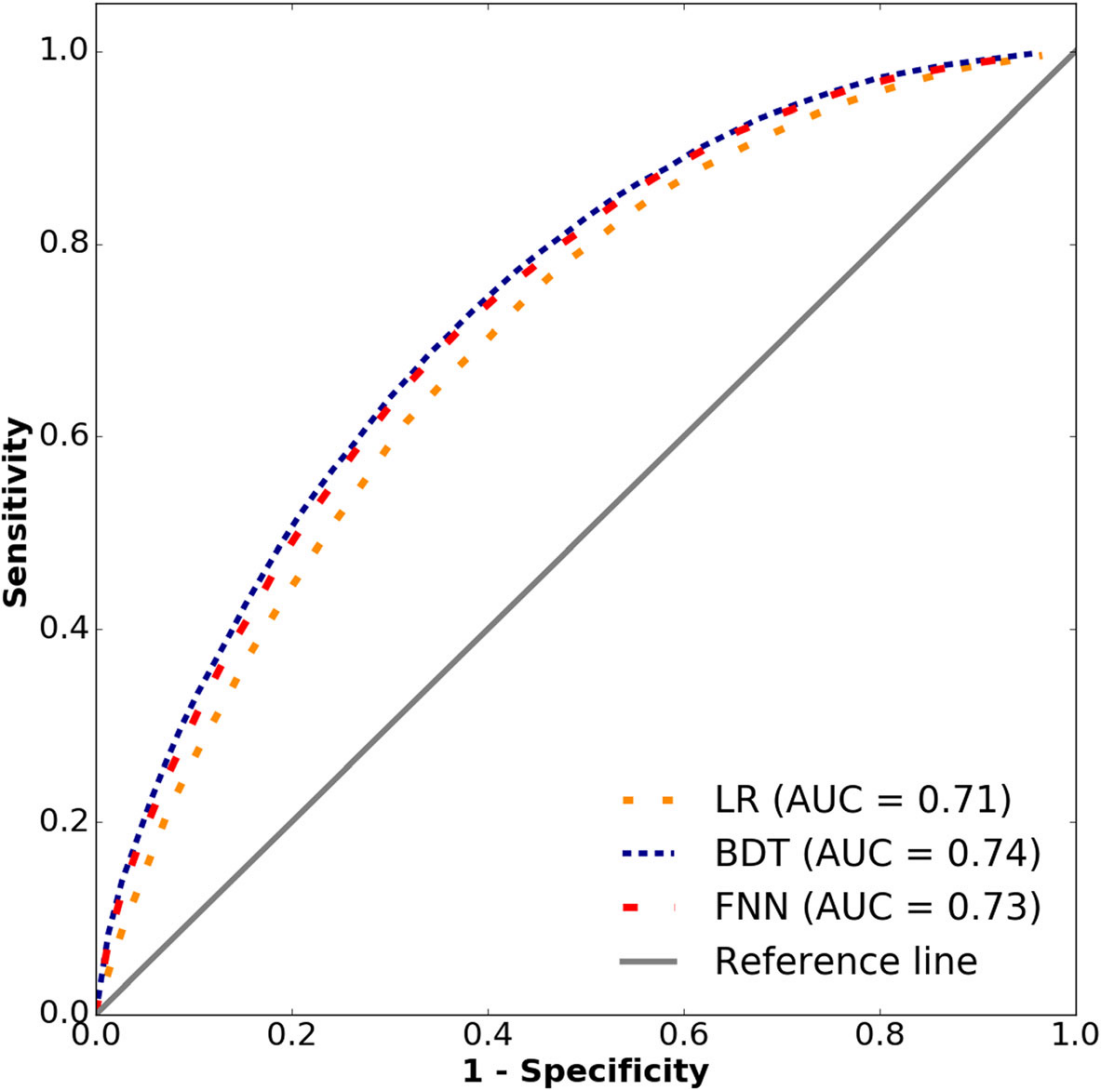
Area under the receiver operating characteristic curve (AUC): Comparison of prediction performance. LR = logistic regression, BDT = boosted decision tree. FNN = feedforward neural network

The BDT model performed the best, leading to 67.6% accuracy and an area under the curve (AUC) score of 0.74, indicating good discrimination between the classes. The receiver operating characteristic curves of all 3 models are presented in Fig. 1. Table 2 indicates the performance of the models on different sets of features. Whereas demographic features alone were not predictive at all, adding simple count measures — especially the number of outpatient office visits and the number of hospitalisations — substantially improved prediction accuracy. The effects of additional features (*n* = 264), total costs, and pharmacotherapy (*n* = 449) were about the same (2–3%), depending on the chosen model. Once combined, the overall accuracy further improved by more than 1%, indicating that these features contain complementary information. As for the model comparison, FNN and BDT consistently outperformed LR by about 2%. Moreover, BDT generalised better on the unseen samples, outperforming the FNN in accuracy by about 0.4%.

### General feature importance

Gradually adding feature sets already provides some intuition about their relative importance, but decision tree models can be further utilised for the systematic analysis of feature importance. Using backward deletion, we found that the number of features could be reduced up to 36, with only a 0.5% loss in the accuracy (Table 2, Additional file 1: Figure S1). We identified the length of hospital stay, total costs, and intravenous mode of drug administration as the most important features. The full list of 36 features is presented in Additional file 1: Table S2. The list comprises both demographic and various medical features such as the number of individual prescriptions, the temporal pattern of outpatient visits, and diabetes as a chronic condition. Interestingly, the following 6 drug classes remained in the model: A03BA (belladonna alkaloids), B03BB (folic acid), N01AH (opioid anaesthetics), N01AX (other general anaesthetics), S01BC (ophthalmologic non-steroidal anti-inflammatory agents) and S01CA (ophthalmologic corticosteroids and anti-infectives in combination).

### Drug importance analysis

#### Conditional drug probabilities

For the total study population, irrespective of prescribed drugs, the probability of cost increase was 51.9%. Conditioned on hospitalisation, the probabilities for increase were 23.1 and 58.1% with and without hospitalisation in 2014, respectively. We subsequently computed the probabilities of increase or decrease in costs conditioned on the 449 drug classes and on hospitalisation. The results are presented in Table 3. In particular, we present the drug classes with the highest probabilities for cost increase or decrease and with frequent prescriptions. All 6 drug classes identified in the previous section are included in this table, with only folic acid (B03BB) being an indicator for an increase in costs.

**Table 3:** Probabilities of cost increase and decrease for patient groups, conditioned on drug groups and hospitalisation

		All		Hospitalisation		No Hospitalisation	
		N	P (Increase), %	N	P (Increase), %	N	P (Increase), %
Total study population (irrespective of drug groups)		373′264	51.9	66′427	23.1	306′837	58.1
**Patients with drug group and highest probability of increase in costs**							
ATC	Name	N	P (Increase), %	N	P (Increase), %	N	P (Increase), %
N06DA	Anticholinesterases	2′214	61.1	587	43.4	1′627	67.4
N04BA	Dopa and dopa derivatives	3′722	56.1	1′158	35.1	2′564	65.6
B01AA	Vitamin K antagonists	18′893	51.6	6′087	27.0	12′806	63.2
B03BB	Folic acid and derivatives	10′467	52.6	3′177	28.4	7′290	63.1
C01BD	Antiarrhythmics, class III	4′622	47.3	1′890	25.7	2′732	62.3
C03CA	Sulfonamides, plain	29′741	50.3	10′793	29.5	18′948	62.1
B03AD	Iron in combination with folic acid	4′483	41.6	1′919	14.8	2′564	61.7
C03BA	Sulfonamides, plain	3′256	52.7	961	31.2	2′295	61.7
G04CB	Testosterone-5a-reductase inhibitors	2′238	53.2	552	27.5	1′686	61.6
A10BB	Sulfonylureas	11′418	54.7	2′298	27.8	9′120	61.4
**Patients with drug group and highest probability of decrease in costs**							
ATC	Name	N	P (Decrease), %	N	P (Decrease), %	N	P (Decrease), %
S01FA	Anticholinergics	2′959	72.4	626	78.9	2′333	70.7
A03BA	Belladonna alkaloids, tertiary amines	7′851	73.4	1′964	82.1	5′887	70.5
N01AH	Opioid anaesthetics	9′715	72.3	2′235	81.9	7′480	69.5
S01HA	Local anaesthetics	7′783	71.5	1′598	79.3	6′185	69.5
A04AA	Serotonin (5HT_3_) antagonists	3′771	70.9	1′637	77.3	2′134	66.0
N01AX	Other general anaesthetics	29′106	68.2	7′299	80.3	21′807	64.1
S01EC	Carbonic anhydrase inhibitors	9′510	66.7	2′120	79.7	7′390	63.0
S01BC	Antiinflammatory agents, non-steroids	13′218	65.3	2′935	77.9	10′283	61.7
C01CA	Adrenergic and dopaminergic agents	13′690	65.3	3′085	79.7	10′605	61.2
A03BB	Belladonna alkaloids, semisynthetic	7′718	66.1	2′089	80.7	5′629	60.8
**Patients with drug group, high probability of decrease in costs and frequent prescriptions**							
ATC	Name	N	P (Decrease), %	N	P (Decrease), %	N	P (Decrease), %
B05BB	Solutions affecting the electrolyte balance	66′792	64.0	20′392	77.9	46′400	58.0
V08AB	Low osmolar X-ray contrast media^*^	29′654	63.7	10′870	78.1	18′784	55.3
S01CA	Corticosteroids and antiinfectives in combination	28′050	59.7	5′774	78.0	22′276	55.0
N01BB	Amides	57′144	59.6	14′887	77.0	42′257	53.5

Bold data are considered table section headings. N = Number of patients per group (characterised by drug group and hospitalisation/no hospitalisation). P (Increase), P (Decrease) = Probability of cost increase or cost decrease in the respective group, displayed in %. The probability of increase in costs conditioned on hospitalisation was computed for all drug classes

Selection criteria: 1.) prescribed to ≥1500 patients who didn’t have a hospitalisation, arranged by descending probability of increase or decrease (top 10 shown), 2.) prescribed to more than 10′000 patients who didn’t have a hospitalisation, arranged by descending probability of increase or decrease (top 4 not included in 1.) shown)

ATC = “Anatomical Therapeutic Chemical” classification code, *Water-soluble, nephrotropic, low osmolar X-ray contrast media

#### Weight analysis

Through the weight analysis, we identified additional drug classes that contributed to the accuracy of prediction. Many of them were found to contribute to predictions of both increases and decreases (Table 4). For instance, magnesium is among the drug groups with a high accuracy for increase (71.4%), but also an important feature for decreases among the patients without hospitalisation (78.6%).

**Table 4:** Weight analysis: Contribution of drug classes to the prediction

**Contribution to prediction of increase**					
ATC	Name	Acc,%	N		
N06DA	Anticholinesterases	77.3	88		
A12CC	Magnesium	71.4	56		
B03BB	Folic acid and derivatives	69.1	408		
A10AE	Insulins and analogues for injection, long-acting	68.1	91		
B01AA	Vitamin K antagonists	67.3	1065		
A03FA	Propulsives	65.5	58		
**Contribution to prediction of decrease**					
		Without Hospitalisation		With Hospitalisation	
ATC	Name	Acc,%	N	Acc,%	N
A12CC	Magnesium	78.6	28	78.8	33
B01AC	Platelet aggregation inhibitors excluding heparin	76.9	26	82.9	345
C01BD	Antiarrhythmics, class III	73.3	30	81.0	58
N06CA	Antidepressants in combination with psycholeptics	69.5	59	75.0	44
B01AA	Vitamin K antagonists	68.8	173	74.5	471
A03FA	Propulsives	65.8	146	84.6	143

All numbers were calculated on the test dataset. Drug groups contributing at least 5% to the overall positive or negative score (complete model without costs). Additionally, the drug classes must have contributed for at least *N* = 40 patients (increase) or *N* = 20 patients (decrease without hospitalisation). The top 6 drug classes are provided, arranged by descending order of accuracy

Acc = Accuracy

N = Number of patients for whom the drug class contributed at least 5% to the overall positive or negative score

ATC = “Anatomical Therapeutic Chemical” classification code

Bold data are significant

### Subgroup analysis

We present examples of the subgroup analysis in Table 5. We found small (100–600 people) but highly predictive subgroups for costs increases (as high as 88%). Moreover, the *gain* because of the drug class was high, reaching up to 23% for folic acid (Example #1) and 21% for oral iron supplements (Example #3). In addition to drug classes, subgroups were further characterised by a variety of features, including outpatient visits, drug prescription information (both counts and temporal information), information on the deductible, home care, and hospitalisation. Example #7 represents a rather large subgroup of patients without hospitalisation that have a high fraction of decrease (fraction of decrease 0.74, gain 18%).

**Table 5:** Examples of subgroups derived from the decision tree

	**Examples of subgroups for increase**	N	PI	Gain
#1	Patients younger than 35 years with at least 1 prescription for folic acid (B03BB), no more than two outpatient office visits in the second quarter of the year, and fewer than 12 drug prescriptions	634	0.88	0.23
#2	Patients with at least 1 prescription for magnesium (A12CC), no hospitalisation (≤ 1 day), at least 5 outpatient office visits with a gynaecologist during the year, no more than 1 outpatient visit in the first quarter of the year overall, and at least 4 visits in the third quarter of the year	265	0.86	0.16
#3	Patients with at least 1 prescription for iron (trivalent, oral preparations, B03AB), a deductible > 1000 Swiss francs for 2014, no change in this deductible for 2015, at least 6 prescribed drugs, and no more than 5 prescriptions*	114	0.78	0.21
#4	Patients with at least 1 prescription for anticholinesterases for dementia (N06DA), no home care (≤ 1 day), no more than 1 prescription in February, and few prescriptions filled by pharmacies	276	0.70	0.13
	**Examples of subgroups for decrease**	N	PD	Gain
#5	Patients with at least 2 prescriptions for anticholinergics for ophthalmologic use (S01FA), no concomitant therapy with Vitamin K antagonists, no more than 11 prescriptions, and no more than 6 outpatient physician visits in the third quarter of the year	303	0.89	0.45
#6	Patients with at least 4 prescriptions for platelet aggregation inhibitors (excluding heparin, B01AC), who were hospitalised (cardiac-related major disease category) and had frequent home care (mean interval < 3.3 days)	3777	0.83	0.04
#7	Patients with at least 2 prescriptions for any other general anaesthetics (N01AX), with a mode of administration ‘intravenously’ and fewer than 2 prescriptions for sulfonamides (C03CA), and no hospitalisation in the first year	4237	0.74	0.18
#8	Patients with at least 1 prescription for both a beta blocking agent (S01ED) and a corticosteroid and anti-infective (S01CA) for ophthalmologic use within one year, who had a maximum of 2 outpatient visits in December	2229	0.67	0.08

N = size of subgroup; PI, PD = conditional probability of increase or decrease for the cuts, P(increase | cut); Gain = |difference P(increase | cut) − P(increase | cut without drug class)|; Maximal number of cuts = 5, *defined as different purchase dates

Bold data are significant

## Discussion

Our models classify patients according to their probability of an increase in costs, with especially a few features contributing substantially to the prediction. Pharmacotherapy provides important information on the cost increase prediction, and its relative importance increases in interaction with other features including health care utilisation. We identified patient subgroups with very high probabilities of increase and decrease.

### Performance of models

Our models predict whether patients’ total health care costs will increase in the subsequent year, with an accuracy of up to 67.6% (AUC 0.74). Lahiri et al. [23] reported a higher accuracy (77.6%) when investigating increases in inpatient claims costs using Medicare data. Although this study is the closest in terms of setting to our study, some major differences should be emphasised: First, Lahiri et al. predicted inpatient expenditures using both inpatient and outpatient information, whereas we consider the change in total health care costs using only outpatient claims and whether or not a patient was hospitalised. Moreover, they found diagnoses and features indicating the development of a new chronic condition the most important features. Diagnoses are not available in our dataset because of legal regulations in Switzerland, and the derivation of features indicating the development of a new chronic condition requires information from the year for which predictions are made. Because these data are typically not available in a prospective scenario, our study was designed so that all the features could be generated without any information from the subsequent year. We found that, for the prediction of a costs increase, medical and costs features contained complementary information. Additionally, the inclusion of medical features facilitates the identification of potential targets for preventive measures [32, 33].

### General feature importance

In general, we found that high healthcare utilisation in the first year was an indicator for a decrease in the following year. Using backward deletion, we identified the 36 most important features, including, for example, length of hospital stay, home care, and count measures for outpatient visits and drug prescriptions. Simple count measures accurately capture the intensity of health care utilisation and therefore may reflect the severity of the disease state [17]. Additionally, when they are generated for multiple timeframes, these measures can be used to introduce valuable temporal information, which was highlighted in a recent study by Morid et al. [28] Interestingly, the counts of drug prescriptions and outpatient visits in the last quarter and the last month of the year are among the most important features, which indicates that the model assigns a risk of therapy continuation in the next year. Intravenously administered drugs are typically associated with some severe conditions, explaining why the intravenous mode of administration was an important feature in our study. Likewise, Pritchard et al. [1] reported that physician-administered injectable or infusible treatments account for a comparably higher fraction of expenditures in high-resource patients. We identified diabetes as an important chronic condition for the prediction of a cost increase, which is consistent with diagnoses identified as important in other studies [23]. In general, chronic conditions [2, 34] and multimorbidity [35] are well-described risk factors for high health care utilisation.

### Drug importance analysis

We found that high probabilities of increase are mainly associated with drug groups used to treat chronic conditions that have a higher likelihood of worsening over time (e.g., anticholinesterases and dopa derivatives for treating dementia or parkinson). In contrast, drug groups associated with higher probability of decrease are predominantly used for severe acute conditions requiring extensive treatment (e.g., adrenergic and dopaminergic agents) or are proxies for expensive procedures, such as (local) anaesthetics used in day surgery. Evaluating the contribution of drug classes to the prediction using a weight analysis, we found that many drug groups contribute to the prediction of both increases and decreases. This finding indicates that the contribution of pharmacotherapy depends on other features and can vary greatly across subgroups.

### Subgroup analysis

When evaluating several example drug groups in more detail, their contribution becomes even clearer. We identified subgroups with a high probability of increase (up to 88%). Although there may be even more, we can derive at least 3 higher-level groups from our examples: 1.) potentially pregnant patients who have not yet delivered; 2.) healthy patients; and 3.) patients suffering from chronic conditions with low use of health care resources. Pregnancy without delivery is considered an important condition for predicting future resource use [36] and is therefore included as a feature in some diagnosis-based comorbidity scores. Lacking diagnosis codes, our model identifies combinations of ATC codes (e.g., folic acid, magnesium), outpatient specialist visits for gynaecology, and few outpatient visits at the beginning of the year as patterns indicating potential pregnancy. For a subgroup of patients hospitalised for delivery, the model predicted a decrease in costs, with as much as 92% accuracy. The “healthy patients” group was characterised by few prescriptions (including at least 1 prescription for oral iron supplements) and a high deductible that did not change in the next year, indicating a self-assessment of very good health status. Self-reported general health has been found to be an important indicator of future health care utilisation in previous studies [18, 37]. Claims data do not include information on self-reported health, so changes in the deductible may serve as an indicator of patients’ individual expectations regarding upcoming health expenditures. Tamang et al. [21] found that patients with a large increase in costs were younger and less likely to have hospitalisation costs and chronic conditions, compared with persistent high-costs patients, which is consistent with our subgroup findings. The final group represents elderly patients suffering from a chronic or worsening conditions, with low use of health care resources, yet having a higher likelihood for an increase in the latter for the following year. Subgroups of patients with a high probability of a cost decrease were characterised by chronic conditions, with intensive health-related claims (hospitalisation, home care), or expensive diagnostic procedures or day surgery.

### Limitations

Change in health care costs is a very broad outcome, and our data represents a whole population, without restrictions on underlying diseases or demographic groups. We therefore found multiple reasons for the increase and decrease in costs, many of which are not predictable or preventable (e.g., accidents). Diagnoses might have provided additional patient information, but they were not available in Swiss claims data. Expensive claims such as hospitalisation in the first year may mask less expensive changes such as new drug prescriptions or additional physician visits in the following year, making the development of costs unsuitable for the evaluation of causal drug-related risk-factors. Model-wise, the main limitation was associated with the sparsity in representing the prescriptions. We think that learning distributed embeddings via techniques similar to skip-gram [38] might mediate this problem. Moreover, it is an active research area to apply recurrent neural networks for learning representations of medical codes and patients [39–43]. In this context, the findings of our study can provide a good starting point for interpreting the results of such advanced models.

### Outlook

This research focused on cost increase on the population level covering two subsequent years. Future research should cover multiple subsequent years. In a recent Danish study, Tamang et al. [21] reported that over the course of eight years, the majority of high-cost patients showed only one high-cost year. Among those with multiple high-cost years, many did not experience them consecutively. In the light of high fluctuation of individual annual costs, the evaluation of an increase in costs using a longer study period may provide insight into long-term effects.

Our project was designed to evaluate the risk factors for cost increase for the total population. While this approach allows for a broad investigation, it naturally reduces the impact of rare drug classes on the overall accuracy. However, such drug classes including chemotherapeutics or biologicals would be of special interest due to their contribution to the overall cost increase in healthcare. To evaluate the impact of rare but high-cost treatments in more detail, future studies have to focus on specific subgroups. This approach would reduce sparsity in the data and would allow to use substances instead of drug classes. Additionally, temporal information on treatment induction, duration and intensity should be included in future analyses.

Our results provide subgroups with high probability of cost increase. This information can help decision makers to optimise the healthcare services for these subgroups through an improved resource allocation planning. For instance, we identified a subgroup of healthy patients which are likely to develop a cost increase. This group may be further investigated with respect to causes, amount and preventability of cost increase. For patients suffering from chronic conditions with low use of health care resources, preventive measures such as disease management programs could be established. Additionally, patients may better choose their deductibles for the next year based on the prediction of the future cost development.

## Conclusion

The development of costs can be predicted using a binary classification. Our results indicate that the contribution of pharmacotherapy depends strongly on other features and can vary across subgroups. Therefore, further studies may focus on the development of models for predefined and therefore less heterogeneous subgroups. The detailed understanding of such subgroups may help to identify potential starting points for improving patient management.

## Supplementary information

**Additional file 1.** Supplementary information: Variance of cost difference explained by basic features using multiple linear regression analysis (**Table S1.** Multiple linear regression models using features observed in 2014) and Backward deletion (**Table S2.** Features included in the small model derived from backward deletion, **Figure S1.** Backward deletion: Number of features included in the complete model and corresponding accuracy levels).

## Data Availability

The datasets analysed during the current study are not publicly available as they are part of the confidential Helsana health insurance claims database. Additional information not included in the paper is available from the corresponding author on reasonable request.

## Abbreviations

ATC: Anatomical Therapeutic Chemical Classification
AUC: Area under the curve
BDT: Boosted decision tree
DRG: Diagnosis-related group
FNN: Feedforward neural networks
GDP: Gross domestic product
GTIN: Global Trade Item Number
LR: Logistic regression
