# Supplementary material for: Prediction of health care expenditure increase: how does pharmacotherapy contribute?
Source: BMC Health Serv Res. 2019 Dec 11;19:953. doi: 10.1186/s12913-019-4616-x (PMC6907182; doi:10.1186/s12913-019-4616-x)
Supplement: Supplementary file 1 — Additional file 1. Supplementary information: Variance of cost difference explained by basic features using multiple linear regression analysis (Table S1. Multiple linear regression models using features observed in 2014) and Backward deletion (Table S2. Features included in the small model derived from backward deletion, Figure S1. Backward deletion: Number of features included in the complete model and corresponding accuracy levels). [file 12913_2019_4616_MOESM1_ESM.docx]

**Supplement**

Variance of cost difference explained by basic features using multiple linear regression analysis

**Statistical Methods**

Because of the skewness of the cost observations, the logarithmic transformation has been applied to assure the approximate normality of the outcome. Multiple linear regression was applied to log(total costs) for 2014 and 2015, and the cost difference was calculated as log(total costs 2015) – log(total costs 2014) to estimate the adjusted coefficient of determination, which describes the explained variance. The computations for the non-transformed costs are also provided to assure comparability with the literature. All models were calculated using R (Version 3.3.1).

**Results**

Using the set of basic features, the explained variance in health care costs, as indicated by the adjusted R-squared value, was considerably smaller for the cost difference than for total costs (basic model: cost difference: 0.2% vs. costs 2014: 3.3% and costs 2015: 2.4%; extended model: cost difference: 7.4% vs. costs 2014: 40.3% and costs 2015: 24.3%). This finding was consistent for all evaluated models (Table S1).

| **TABLE S1.** Multiple linear regression models using features observed in 2014 | | | | | | |
| --- | --- | --- | --- | --- | --- | --- |
| **Outcome** | | | | | | |
|  | **Costs**  **2014** | **Log(Costs) 2014** | **Costs**  **2015** | **Log(Costs) 2015** | **Cost Difference**  **2015–2014** | **Log(Cost Difference) 2015–2014** |
| **Features** | adj. R^2^ | adj. R^2^ | adj. R^2^ | adj. R^2^ | adj. R^2^ | adj. R^2^ |
| age + gender | 0.021 | 0.041 | 0.030 | 0.052 | 0.002 | 0.001 |
| age + gender + area of residence | 0.022 | 0.043 | 0.031 | 0.054 | 0.002 | 0.001 |
| age + gender + area of residence + deductible | 0.027 | 0.060 | 0.036 | 0.070 | 0.002 | 0.001 |
| **Demographic model*** | 0.033 | 0.068 | 0.042 | 0.077 | 0.002 | 0.001 |
| + number of different drugs  + number of individual prescriptions | 0.243  0.284 | 0.391  0.337 | 0.134  0.186 | 0.220  0.237 | 0.020  0.010 | 0.031  0.022 |
| + number of hospitalisations | 0.234 | 0.261 | 0.099 | 0.149 | 0.057 | 0.010 |
| + number of outpatient physician office visits | 0.183 | 0.345 | 0.093 | 0.173 | 0.022 | 0.020 |
| + chronic conditions | 0.228 | 0.275 | 0.156 | 0.208 | 0.016 | 0.022 |
| **Extended model**** | 0.403 | 0.459 | 0.243 | 0.306 | 0.074 | 0.043 |

*Demographic model = age + gender + area of residence + deductible + insurance model

**Extended model = Demographic model + number of different drugs + number of individual prescriptions + number of hospitalisations + number of outpatient physician office visits + chronic conditions

Backward deletion: Included features

| **TABLE S2.** Features included in the small model derived from backward deletion | |
| --- | --- |
| **Feature name** | |
| 1 | Length of hospital stay per year |
| 2 | Total health care costs in 2014 |
| 3 | Mode of drug administration: intravenous |
| 4 | Number of hospitalisations |
| 5 | Drug group: S01CA (ophthalmologic corticosteroids and anti-infectives in combination) |
| 6 | Drug group: N01AX (other general anesthetics) |
| 7 | Age (categorical variable) |
| 8 | Number of individual drug prescriptions (GTINs) filled in the last quarter of the year |
| 9 | Length of stay in inpatient psychiatric facility per year |
| 10 | Number of days of home care (including basic care, treatments, and administration) |
| 11 | Number of hospitalisations: major diagnostic category — muscular–skeletal-related diseases |
| 12 | Number of outpatient physician office visits |
| 13 | Drug group: B03BB (folic acid) |
| 14 | Number of hospitalisations: major diagnostic category — delivery |
| 15 | Number of outpatient physician office visits: physician specialisation — gynaecologist |
| 16 | Number of outpatient physician office visits: physician specialisation — gastroenterologist |
| 17 | Number of outpatient physician office visits in December |
| 18 | Deductible |
| 19 | Change of chosen deductible in the next year |
| 20 | Drug group: N01AH (opioid anesthetics) |
| 21 | Number of outpatient physician bedside visits in the last quarter of the year |
| 22 | Length of nursing home stay per year |
| 23 | Drug group: S01BC (ophthalmologic non-steroidal anti-inflammatory agents) |
| 24 | Gender |
| 25 | Drug group: A03BA (belladonna alkaloids) |
| 26 | Length of stay in rehabilitation facility per year |
| 27 | Number of days of home care (basic care only) |
| 28 | Number of individual drug prescriptions (GTINs) |
| 29 | Number of outpatient physician office visits in the last quarter of the year |
| 30 | Number of outpatient physician office visits: physician specialisation — ophthalmologist |
| 31 | Median interval between outpatient physician office visits |
| 32 | Number of individual drug prescriptions (GTINs) filled in December |
| 33 | Chronic condition: diabetes mellitus |
| 34 | Individual drug prescriptions (GTINs) dispensed by hospitals |
| 35 | Number of outpatient physician office visits in the first quarter of the year |
| 36 | Number of invoices for home care (including basic care, treatments, and administration) |

Most important 36 features, arranged in descending order of loss reduction. GTIN = Global Trade Item Number, which was used to identify prescribed drugs

Backward deletion: Process


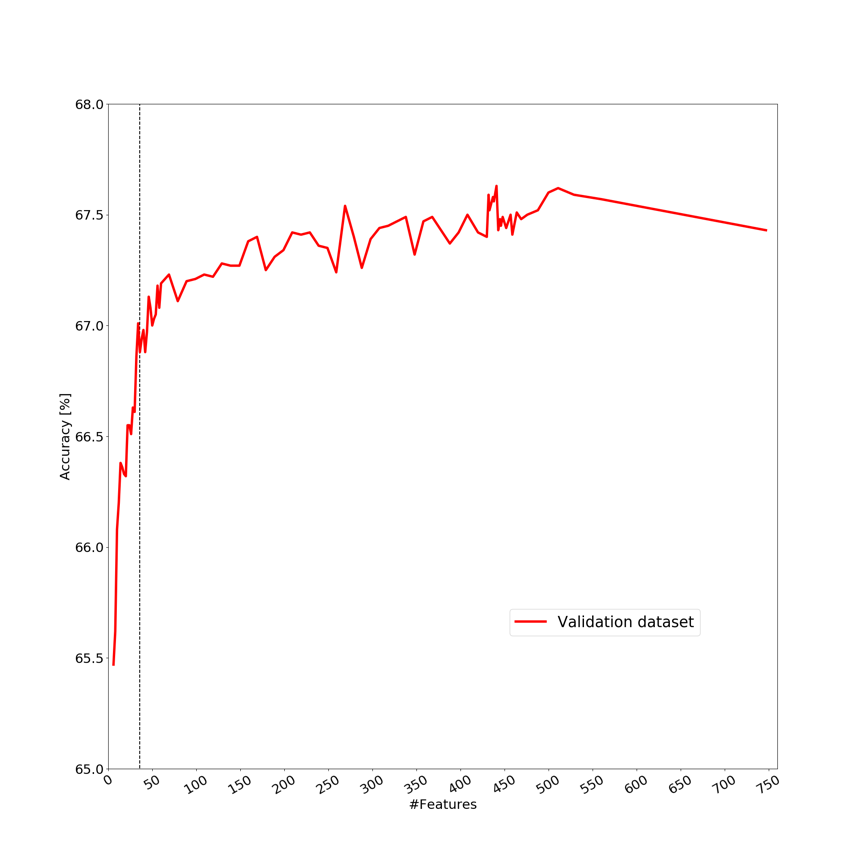


**Figure S1. Backward deletion: Number of features included in the complete model (boosted decision tree, 747) and corresponding accuracy levels.** The vertical black line represents the 36 most important features.
